# Data on the intermolecular interactions of 1,1,1,2-tetrafluoroethane liquids from molecular dynamics simulations

**DOI:** 10.1016/j.dib.2023.109485

**Published:** 2023-08-11

**Authors:** Chin W. Yong, Vivian Walter Barron, Alex Slowey, Ilian T. Todorov, Kevin J. Roberts, Robert B. Hammond

**Affiliations:** a Scientific Computing Department, Science and Technology Facilities Council, Daresbury Laboratory, Sci-Tech Daresbury, Warrington, WA4 4AD, UK; b Division of Pharmacy and Optometry, the University of Manchester, Manchester, M13 9PL, UK; c School of Chemical and Process Engineering, University of Leeds, Leeds, LS2 9JT, UK; d Kindeva Drug Discovery, Loughborough, Leicestershire, LE11 5RB, UK

**Keywords:** HFA-134a, Atomic interactions, Molecular dynamics, DL_ANALYSER, DANAI, Hydrogen bonds, Induced-dipole, Halo-alkanes

## Abstract

Detailed atomistic interactions of 1,1,1,2-tetrafluoroethane (HFA-134a) liquid were presented in a data format, namely, DL_ANALYSER Notation for Atomic Interactions (DANAI), that annotates precisely the nature of interactions that is discoverable and searchable without having to resolve to diagrammatic illustrations. The datasets were obtained from raw atomic trajectory files of HFA-134a pure liquid models produced by using DL_POLY molecular dynamics software package. The trajectory datafiles contain expressions of atomic species in a natural chemical sense, and hence, provide localized key interactions, ‘at a glance’, of the liquid model on otherwise a typically disordered system consists of complex network of intermolecular interactions. The data provide insights to detailed structural behavior of molecules in liquid phase, and can be used as cheminformatics comparative investigations, linking to other molecular system models that contain similar interaction types and chemical species. This can form the foundation of investigations into the role of HFA-134a plays within different applications. For example, it can be used to compare structural and atomic interaction differences with alternative refrigerants, or as liquid propellants in pharmaceutical devices when solvating formulation ingredients.

**Specifications Table**

**Table:** 

Subject	Physical Sciences
Specific subject area	Computational chemistry in areas of classical molecular simulations.
Type of data	Table Figure
How the data were acquired	Molecular dynamics (MD) simulations were carried out by using DL_Software, a collective term for the computational chemistry software developed at the STFC, Daresbury Laboratory. Three independent DL_Software components – DL_FIELD, DL_POLY and DL_ANALYSER, have been used to generate the data, which formed an integrated software infrastructure for carrying out molecular simulations. An HFA-134a force field model represents the liquid system was set up by using DL_FIELD 4.9. A series of MD simulations were performed by using DL_POLY 4.10 software on the High-Performance Computing center at the University of Leeds, UK, over a range of temperatures (203 K – 323 K). At each temperature setting, a series of atomic trajectory frames were written out to a file (raw trajectory data). After that, the trajectory data was analyzed by using DL_ANALYSER 2.2 to produce the atomic interaction data.
Data format	Raw. Analyzed.
Description of data collection	MD simulations were performed to iteratively calculate atomic positions based on the classical forces exert on each atom. The atom positions were periodically written out to a file during the calculation process to produce a raw trajectory data file. Based on these atoms’ positions, the DL_ANALYSER software was used to identify the induced-dipole (ID) interactions, based on a distance criterion between two non-bonded carbon centers of the molecules. After that, DL_ANALYSER classified these interactions into various modes that indicate the nature and extent of such interactions and their local topological arrangements. These interaction modes were subsequently accumulated and averaged over all trajectories.
Data source location	Institution: University of Leeds, Country: UK
Data accessibility	Repository name: eData: the STFC Research Data Repository Data identification number: 10.5286/edata/905 Direct URL to data: https://edata.stfc.ac.uk/handle/edata/936
Related research article	V.W. Barron, C.W. Yong, A. Slowey, I.T. Todorov, K.J. Roberts, R.B. Hammond, Comparison between the intermolecular interactions in the liquid and solid forms of propellant 1,1,1,2-tetrafluoroethane, J. Mole. Liq. https://doi.org/10.1016/j.molliq.2023.121993

## 1 Value of the Data

• The atomic trajectory data contained a complete simulated information about structure and interaction behavior of HFA-134a molecules in liquid phase over a range of temperature.
• Analysis of atomic interactions for HFA-134a molecules, highlighting the extent and nature of ID interactions and their correlations.
• Provide an overall view of the atomistic structures of the molecules for a disordered system in condensed phase by annotating the interaction behavior in a novel syntax which can be interpreted by cognitive means without resorting to detailed pictorial or diagrammatic illustrations.
• Enable atomic interactions to become data accessible and discoverable by computational means.
• The chemical-sensitive data can be used for comparative studies with respect to other results data that also contain atomistic information and be useful in cheminformatics on predictions and statistical model constructions for molecular systems.

## 2 Objective

HFA-134a is a hydrofluorocarbon that found its use as a pressurized liquid propellant in metered dose inhaler formulations, which mixed well with the active pharmaceutical ingredients (APIs). To produce a reliable and consistent API dosage, detailed knowledge about solvent structures and the nature of its interactions with APIs is important in product formulations. To this end, a series of MD simulations had been carried out [1] to provide atomistics insights into the structural behavior of the HFA-134a liquid propellent, which is not accessible by experimental means. Detailed geometrical and orientational structures of the liquid had been rationalized and elucidated based on the secondary analysis of the atomic interaction data reports here.

In this paper, the datasets provide a more extensive view to the overall behavior of the molecular structures at various temperatures. Since MD simulations essentially contain a complete range of atomistic interactions, it would be useful to reduce the inherently rich and complex information into a tractable view of the molecular system. In addition, correlational information is also included in the datasets, to provide detailed information on the characteristic behavior of various modes of interactions identified in the system, and how they are related to one another.

## 3 Data Description

The data repository consists of a number of raw data trajectory files each contained atom labels and the corresponding atomic coordinates of HFA-134a liquid simulation models produced by DL_POLY version 4.10 MD simulation software [2]. The atom labels were expressed in DL_F Notation [3], to enable atomic interaction analysis to be carried out by using DL_ANALYSER software [4]. Five simulations had been run, each at a different temperature: 203 K, 233 K, 263 K, 293 K and 323 K. Each simulation dataset is stored in a separate file in the compressed (gzipped) ASCII text format, with the general filename *HISTORY_XXK.gz*, where *XX* refers to the temperature of the simulation runs.

The repository also contains a set of analyzed data obtained from the raw trajectory data, which describes various types of atomic interactions with respect to MD simulation time. The raw data files were analyzed by using DL_ANALYSER version 2.2 software and the various types of interactions were identified from the simulation trajectories and transcribed into an expression syntax called DL_ANALYSER Notation for Atomic Interaction (DANAI) [4]. The process was carried out for all the interactions listed in Table 1 for all trajectory frames that corresponds to the MD time from 0 ns up to 5 ns.

**Table 1:** List of different modes of atomic interactions expressed in DANAI statements, indicating the nature and extent of interactions.

(A) ID_182_182	(B) ID_180_180	(C) ID_180_182
1. [L2]C182:C182	1. [L2]C180:C180	1. [L2]C182:C180
2. [L2]c182:c182	2. [L2]c180:c180	2. [L2]c182:c180
3. [L3]c182:c182:c182	3. [L3]c180:c180:c180	3. [L3]c182:c180:c182
4. [R3]c182:c182:c182:c182	4. [R3]c180:c180:c180:c180	4. [R3]c182:c180:c182:c182
5. [J4]c182:c182(:c182):c182	5. [J4]c180:c180(:c180):c180	5. [L3]c180:c182:c180
6. [J4]C182:C182(:C182):C182	6. [J4]C180:C180(:C180):C180	6. [R3]c180:c182:c180:c180
7. [J5]c182:(c182:)c182(:c182):c182	7. [J5]c180:(c180:)c180(:c180):c180	

**Fig. 1:**
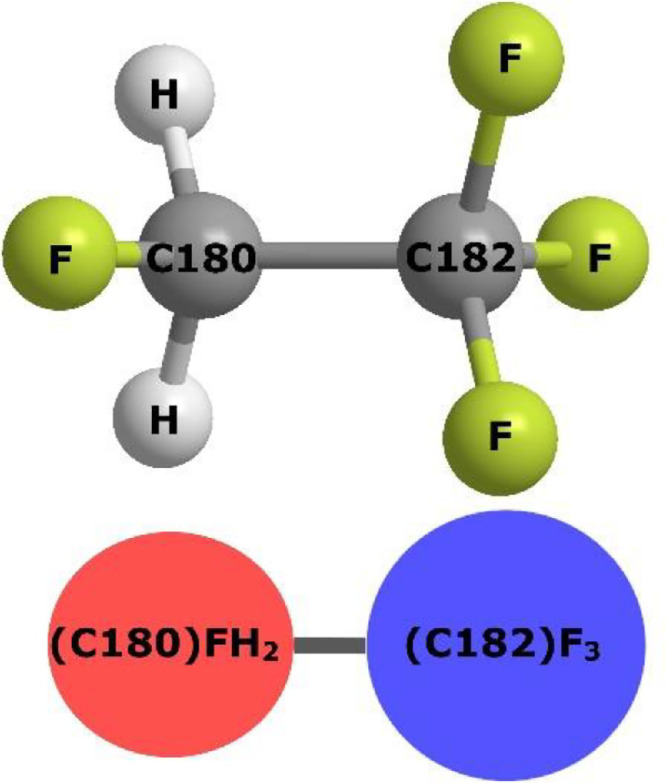
Ball-and-stick representation of HFA-134a, along with the schematic representation as blue and red spheres, centered around C180 and C182, respectively.

Detailed instructions how to interpret DANAI have been described elsewhere [4], but due to the novelty of the Notation, this is described in more details in terms of HFA-134a molecules as follows:

Fig. 1 shows a ball-and-stick representation of the HFA-134a molecule, showing the elemental symbols fluorine and hydrogen atoms. For the carbon atoms, they are labelled with the DL_F Notation, where the unique numerical values of 180 and 182 indicate they are of the *monohaloalkane* and *trihaloalkane* type, respectively. The HFA-134a molecule can be categorized into two different groups centered at the respective carbon atoms: the monofluoroalkyl group represented by a red sphere, and trifluoroalkyl group represented by a blue sphere.

The molecules are predominantly interacting with one another via the intermolecular induced-dipole (ID) interactions and the significance of such interactions are identified based on the distance criteria (5 Å) between any two non-bonded spheres centered round the carbon atoms. Although ID interactions can occur over longer range, a small value of 5 Å was used to ensure only close contacts are considered and there is no other molecule straddle in between the two carbon centers. From such, a global interaction map of HFA-134a was constructed for each trajectory frame, that allows DL_ANALYSER to identify and count the number of a similar interaction pattern. These interaction patterns are then transcribed into the DANAI syntax.

A DANAI expression consists of a set of symbols, including the atomic (chemical) species represented by the DL_F Notation that describes the actual chemical identity of atoms in the system.

Of various interactions identified among the molecules, the formers can be classified into three different ID *macro-interactions* within the context of DANAI: ID_182_182, or ‘only among blue spheres’; ID_180_180, or ‘only among red spheres’; and ID_180_182, or ‘among the red and blue spheres’. For each macro-interaction, there are various modes of interactions, or the *micro-interactions* that indicate the different nature and extent of interactions among the participating chemical species, as indicated by the macro-interaction, that can possibly occur. DL_ANALYSER only analyzed a certain subset of these interactions, which are confined to local levels, involving only a few chemical species, as shown in Table 1.

**Fig. 2:**
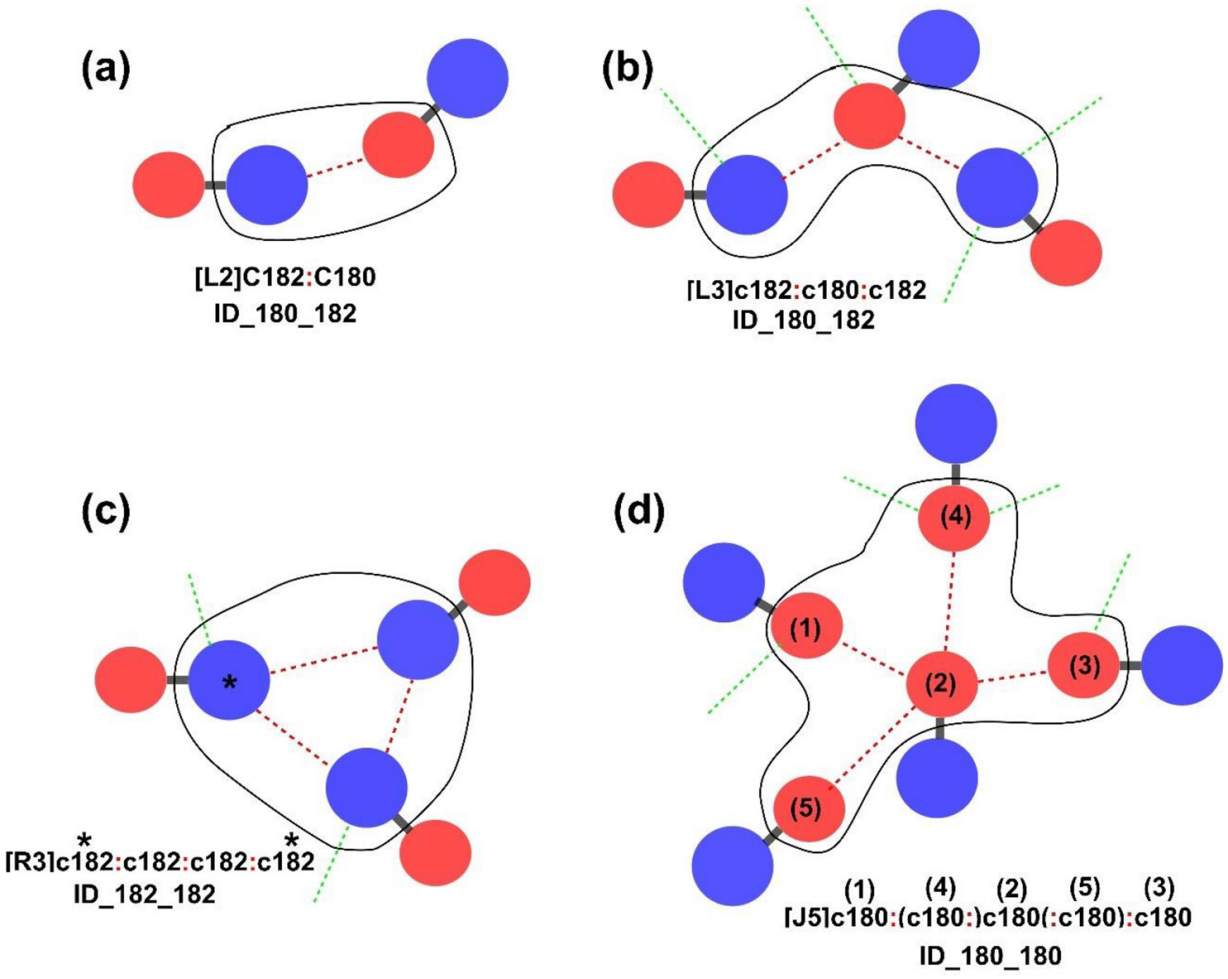
Some examples of micro-interaction statements with the corresponding diagrammatic illustrations. The red dotted lines refer to the ID interactions, shown by the symbols: in the DANAI statements. The green dotted lines refer to possibly some other ID interactions involving other chemical species (not shown) as indicated in the macro-interactions. Black lassos sketch the region of interacting species according to the DANAI statements

Fig. 2 shows four examples of micro-interaction statements with the corresponding diagrammatic illustrations. The interpretations of the DANAI statements are summarized as follows:

(1) The information contains within the square brackets indicates the topological structure and the number of participating chemical species that formed such a structure in the interaction. In these cases, L refers to linear, R refers to ring and J refers to junction. For example, [L3] means three chemical species interacting serially, forming a linear structure, Fig. 2(b).
(2) The colon (:) represents the non-bonded interaction between two chemical species. This is shown as the red dotted lines in Fig. 2 examples.
(3) For interactions forming a ring structure, the first and the last chemical species refer to the same species, which indicates the extent of the ring enclosure. For instance, the chemical species marked with * in Fig. 2(c), which corresponds to the marked DANAI statement.
(4) A chemical species enclosed within a bracket means it is a branched species. For instance, consider that species (1), (2) and (3) are interacting with one another, forming a linear interacting chain, as shown in Fig. 2(d). Chemical species that are labelled (4) and (5) are regarded as the two branched species that interact with the member species along the chain. Subsequently, they are enclosed within brackets in the DANAI statement. Of note are the locations of species (4) and (5) and their corresponding: symbols that are positioned in the statement. These symbols indicate which member species located along the chain they are interacting with. The DANAI statement indicates both species (4) and (5) are interacting with species (2). For example, species (4) is placed between species (1) and species (2) in the DANAI statement. The: symbol within the bracket is placed to the right, to indicate the interaction is with species (2) and not species (1). Similarly, species (5) is placed between species (2) and species (3). The: symbol within the bracket is placed to the left, to indicate species (5) interacts with species (2) and not species (3).
(5) If a chemical species is specified in the DANAI statement in the uppercase letter, it means the only ID interaction involve with this species is what is indicated in the micro-interaction statement. For example, the DANAI statement in Fig. 2(a) is expressed as ‘**C**182:**C**180’. Since both are expressed in capital letter ‘C’, this means the only detected ID interaction at the C182 species is with a C180 species and *vice-versa*. No additional ID interaction is detected with other chemical species (as with other C180 or C182). In other words, it is an isolated interacting pair of chemical species.
(6) If a chemical species is specified in the DANAI statement in the lowercase letter, it means the chemical species may interact with more than one other chemical species, including those species that are not shown in the statement. These additional ID interactions are shown as green dotted lines in Fig. 2. Consider Fig. 2(c) for ID_182_182, where three C182 species interact with one another, forming a ring structure. These species are expressed as ‘**c**182’, implying that there may be other ID interactions detected with other C182 species, as indicated by the green dotted line, apart from the said species that involved in the ring formation.

**Fig. 3:**
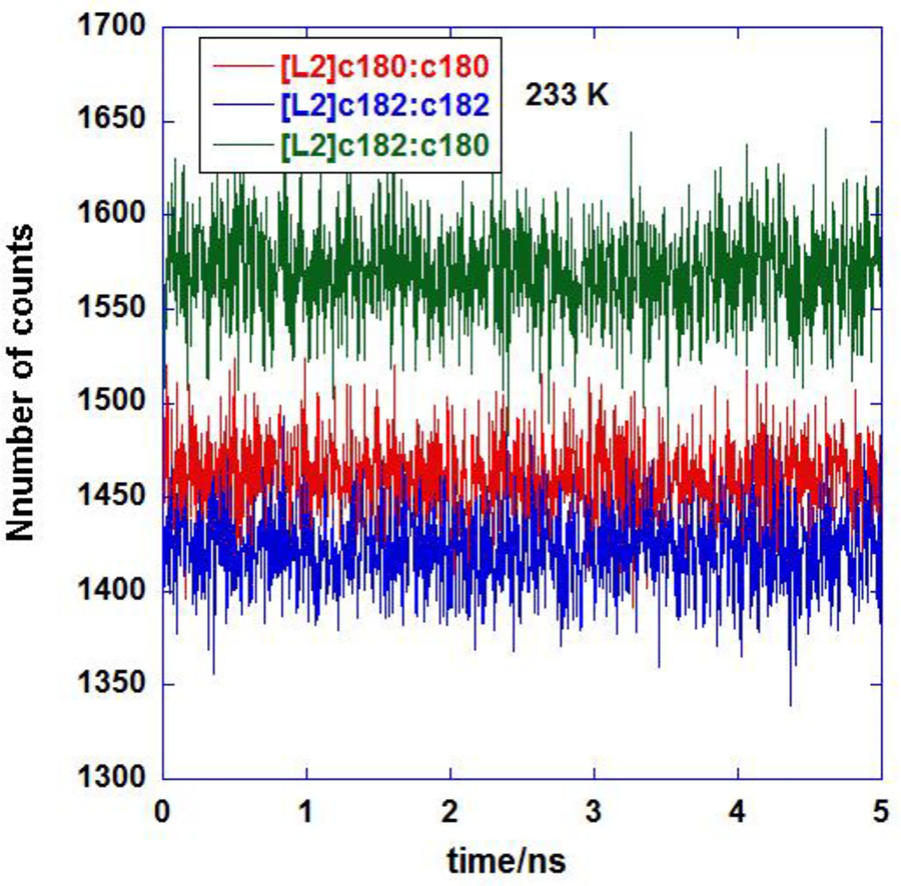
Time variation profiles for [L2]c180:c180, [L2]c182:c182 and [L2]c182:c180 at 233 K. Data extracted from *DANAI_HFA-134a_233K.xlsx.*

The analyzed DANAI data are listed in five separate Excel files, one for each temperature as mentioned above. The filenames are *DANAI_HFA-134a_XXK.xlsx* where *XX* refer to the temperature of simulation run. Within each file, it contains lists of count values for each MD time frame that correspond to the interaction modes as shown in Table 1. The dataset can be plotted to show the time profile variation of atomic interactions. For example, Fig. 3 shows the time profile variations for interaction modes [L2]c180:c180, [L2]c182:c182 and [L2]c182:c180 at 233 K.

In addition, the overall interaction behavior of the molecules has also been obtained from DL_ANALYSER by calculating the average count, *μ*, over all time frames for each interaction mode and their inter-relationships can be accessed by determining the correlation of coefficients, $C_{x-y}$, between any two interaction modes *x* and *y*.

$$C_{x-y}=\frac{\langle \Delta C_{x}\cdot \Delta C_{y}\rangle }{\sqrt{\langle \Delta C_{x}^{2}\rangle \langle \Delta C_{y}^{2}\rangle }}$$

$$\Delta C_{i}=C_{i}-\mu_{i}$$

These datasets are stored in Excel form in the file *DANAI_HFA-134a_average_correlation.xlsx* for all temperatures. For example, Table 2 shows the average values of all identified interactions at 263 K, which were extracted from the Excel sheet labelled as ID_182_182 in the file.

**Table 2:** Average values of ID_182_182 interactions and the standard deviations.

ID_182_182	Average counts	Deviation
1. [L2]C182:C182	45.0784	6.916896
2. [L2]c182:c182	1264.3432	21.657549
3. [L3]c182:c182:c182	2320.4	78.639603
4. [R3]c182:c182:c182:c182	330.8568	34.426099
5. [J4]c182:c182(:c182):c182	402.3512	16.327469
6. [J4]C182:C182(:C182):C182	0.1512	0.400423
7. [J5]c182:(c182:)c182(:c182):c182	113.6656	9.862585

Table 3 shows the corresponding correlation coefficients between different pairs of interactions at 263 K. The value of $C_{x-y}$ is obtained as the intersection of the interaction values *x* and *y* shown at the top row and left column of the table. The interaction values, which ranged from 1 to 7, corresponds to the numerical labels in Table 2. For example, $C_{3-5}$ = 0.778, which show the correlation extent between the interactions [L3]c182:c182:c182 and [J4]c182:c182(:c182):c182.

**Table 3:** Correlation coefficients between interaction *i* and *j* with the values from 1 to 7, which corresponds to the numerical sequence shown in Table 2.

263 K	1	2	3	4	5	6	7
1	1.000	−0.405	−0.348	−0.164	−0.273	0.082	−0.160
2		1.000	0.923	0.612	0.716	−0.031	0.527
3			1.000	0.444	0.778	−0.013	0.716
4				1.000	0.121	0.014	0.119
5					1.000	−0.020	0.469
6						1.000	0.018
7							1.000

## 4 Experimental Design, Materials and Methods

The DL_Software computational chemistry software suite, namely, DL_FIELD, DL_POLY and DL_ANALYSER, were used in tandem to carry out molecular dynamic simulations.

DL_FIELD was used to set up the initial liquid molecular configuration system and the force field model employed is an OPLS [5] variant of the force field scheme that is fitted specifically to model HFA-134a [6]. The chemical-sensitive DLF_Notation [3] was specified in the DL_FIELD control file for the atom labels, to ensure interaction analysis can be carried out on simulation outputs. To construct the liquid model, a single HFA-134a molecule was first constructed by using the Chem3D [7] package as the initial input configuration for DL_FIELD. Then, the Solution Maker feature contained within DL_FIELD was used to duplicate 1000 molecules in random orientations and enclosed in a periodic cubic box with an initial length of 53 Å. For detailed, stepwise procedures to set up a liquid model, please consult DL_Software Digital Guide site [8].

To carry out MD simulations in DL_POLY, the van der Waals and coulombic real space cut off were set to 14 Å. The coulombic interactions were treated by means of SPME [9] with the ewald precision parameter set to 10^−6^. The initial liquid system was equilibrated at the NVE ensemble, with the atoms’ velocities rescaled to a given temperature, from 10 K and gradually increased until the target temperature is reached. Once the system was found to maintain a steady target temperature and configuration energy even without velocity rescaling, the ensemble was switched to the NPT and the Nosé-Hoover formalism [10] was used to maintain a constant temperature and the pressure at 20 atm. The thermostat and barostat constants were set to 0.4 ps and 1.0 ps, respectively. The system was equilibrated for a further 1 ns at NPT until the box size has attained a steady value. This was then followed by the sampling process at the NVT ensemble for a total of 5 ns by using a fixed MD time-step of 2 fs. During the sampling run, a series of atomic trajectory frames were written out to a file at every 4 ps (2000 MD steps). The procedures were repeated for a different target temperature, to produce the corresponding trajectory files.

Finally, DL_ANALYSER software was used to carried out the atomic interaction analysis, by setting 5 Å critical distance between two non-bonded carbon atoms as the criterion to produce a global interaction map within each trajectory frame.

## Ethics Statements

All data acquire in this work are purely digital in nature and does not involve in life subjects.

## CRediT authorship contribution statement

**Chin W. Yong:** Conceptualization, Methodology, Software, Data curation, Writing – original draft. **Vivian Walter Barron:** Investigation, Writing – review & editing. **Alex Slowey:** Funding acquisition, Project administration. **Ilian T. Todorov:** Software, Writing – review & editing. **Kevin J. Roberts:** Writing – review & editing. **Robert B. Hammond:** Supervision, Writing – review & editing.

## Declaration of Competing Interest

The authors declare that they have no known competing financial interests or personal relationships that could have appeared to influence the work reported in this paper.

## Data Availability

Molecular simulation trajectories and atomic interaction descriptions of 1,1,1,2-tetrafluoroethane liquids (Original data) (STFC eData).

## References

[1] Barron V.W., Yong C.W., Slowey A., Todorov I.T., Roberts K.J., Hammond R.B. (2023). Comparison between the intermolecular interactions in the liquid and solid forms of propellant 1,1,1,2-tetrafluoroethane. J. Mol. Liquids.

[2] Smith W., Yong C.W., Rodger P.M. (2002). DL_POLY: application to molecular simulation. Mol. Sim..

[3] Yong C.W. (2016). Description and implementations of DL_F Notation: A natural chemical expression system of atom types for molecular simulations. J. Chem. Inf. Model..

[4] Yong C.W., Todorov I.T. (2018). DL_ANALYSER Notation for Atomic Interactions (DANAI): A natural notation system for molecular interactions, using ethanoic acid liquid as a test case. Molecules.

[5] Jorgensen W.L., Maxwell D.S., Tirado-Rives J. (1996). Development and testing of the OPLS all-atom force field on conformational energetics and properties of organic liquids. J. Am. Chem. Soc..

[6] Peguin R.P.S, Kamath G., Potoff J.J., da Rocha S.R.P. (2009). All-atom force field for the prediction of vapor-liquid equilibria and interfacial properties of HFA134a. J. Phys. Chem. B.

[7] Chem3D version 13.0, Revvity Signals Software.

[8] Detailed procedures to set up a liquid model for DL_POLY molecular simulations, at DL_SDG site: https://dl-sdg.github.io/RESOURCES/SIMULATIONS/sample_prep.html.

[9] Essmann U., Perera L., Berkowitz M.L., Darden T., Lee H., Pedersen L.G. (1995). A smooth particle mesh Ewald method. J. Chem. Phys..

[10] Melchionna S., Ciccotti G., Holian B.L. (1993). Hoover NPY dynamics for systems varying in shape and size. Mol. Phys..

